# Haplotypes and Sequence Variation in the Ovine Adiponectin Gene (*ADIPOQ*)

**DOI:** 10.3390/genes6041230

**Published:** 2015-11-23

**Authors:** Qing-Ming An, Hui-Tong Zhou, Jiang Hu, Yu-Zhu Luo, Jon G. H. Hickford

**Affiliations:** 1 Gansu Key Laboratory of Herbivorous Animal Biotechnology, Faculty of Animal Science and Technology, Gansu Agricultural University, Lanzhou 730070, China; E-Mails: anqingming2009@163.com (Q.-M.A.); Zhou@lincoln.ac.nz (H.-T.Z.); huj@gsau.edu.cn (J.H.); 2 Gene-Marker Laboratory, Faculty of Agriculture and Life Sciences, Lincoln University, Lincoln 7647, New Zealand

**Keywords:** adiponectin, *ADIPOQ*, variation, haplotype, PCR-SSCP, sheep

## Abstract

The adiponectin gene (*ADIPOQ*) plays an important role in energy homeostasis. In this study five separate regions (regions 1 to 5) of ovine *ADIPOQ* were analysed using PCR-SSCP. Four different PCR-SSCP patterns (*A_1_-D_1_*, *A_2_-D_2_*) were detected in region-1 and region-2, respectively, with seven and six SNPs being revealed. In region-3, three different patterns (*A_3_-C_3_*) and three SNPs were observed. Two patterns (*A_4_-B_4_*, *A_5_-B_5_*) and two and one SNPs were observed in region-4 and region-5, respectively. In total, nineteen SNPs were detected, with five of them in the coding region and two (c.46T/C and c.515G/A) putatively resulting in amino acid changes (p.Tyr16His and p.Lys172Arg). In region-1, -2 and -3 of 316 sheep from eight New Zealand breeds, variants *A_1_*, *A_2_* and *A_3_* were the most common, although variant frequencies differed in the eight breeds. Across region-1 and region-3, nine haplotypes were identified and haplotypes *A_1_-A_3_*, *A_1_-C_3_*, *B_1_-A_3_* and *B_1_-C_3_* were most common. These results indicate that the *ADIPOQ* gene is polymorphic and suggest that further analysis is required to see if the variation in the gene is associated with animal production traits.

## 1. Introduction

As a member of the adipocytokine family, adiponectin (ADIPOQ) is secreted mainly by the white adipose tissue, but it can also be produced by other tissues such as brown adipose tissue, bone marrow and skeletal muscle [1,2,3]. It has been demonstrated that ADIPOQ plays an important role in the adipokine signalling pathway and thus it can regulate energy homeostasis, glucose metabolism and lipid metabolism [4]. In humans and mice, the expression of *ADIPOQ* in adipose tissue is negatively correlated with obesity [5].

The human adiponectin gene (*ADIPOQ*) was first identified in 1995 and is located on human chromosome 3q27 [6,7]. It spans about 17 kb and contains three exons and two introns. Variation in the human gene has been reported to be associated with obesity, type 2 diabetes susceptibility, cancer risk, serum adiponectin levels and Chronic Obstructive Pulmonary Disease [8,9,10,11]. In livestock species, polymorphisms in *ADIPOQ* have been associated with various traits including chest circumference in goats [12], fat deposition, carcass traits and reproduction traits in pigs [13,14], and meat marbling, ribeye muscle area and carcass fat thickness in cattle [3]. Despite these findings the various functions of *ADIPOQ* remain poorly understood, but the reports published to date suggest that *ADIPOQ* may be an important gene for key animal production traits.

In this study, the objective was to search for polymorphism in different regions (spanning the promoter region to the 3'-UTR region) of *ADIPOQ* in different breeds of sheep, in anticipation of a much larger study to ascertain whether the variation has an effect on ovine growth, carcass and other production traits.

## 2. Materials and Methods

All research involving animals was carried out in accordance with the Animal Welfare Act 1999 (New Zealand Government) and the collection of sheep blood drops by nicking sheep ears is covered by Section 7.5 Animal Identification, of the Animal Welfare (Sheep and Beef Cattle) Code of Welfare 2010; a code of welfare issued under the Animal Welfare Act 1999 (New Zealand Government).

### 2.1. Sheep Investigated and DNA Collection

One hundred unrelated sheep, selected from a variety of common breeds in New Zealand (NZ) including Merinos (*n* = 18), Suffolks (*n* = 21), Texels (*n* = 22), Dorset Downs (*n* = 19) and NZ Romneys (*n* = 20), were used initially to screen for variation in *ADIPOQ* by methods that are described below. Having identified that variation existed in these sheep, an additional 216 unrelated sheep from a variety of breeds were added to the analysis to enable more accurate determination of individual variant frequencies.

Samples of blood were collected directly onto FTA cards (Whatman BioScience, Middlesex, UK) and DNA for analysis was purified from the dried blood spots, using a procedure described by Zhou *et al*. [15].

### 2.2. PCR Amplification and SSCP Analysis

Five different regions of ovine *ADIPOQ* located at or near the coding sequences were chosen for analysis. These were region-1 (containing part of the promoter, entire exon 1 and part of intron 1), region-2 (containing entire exon 2 and flanking sequences from introns 1 and 2), region-3 (containing part of intron 2 and part of exon 3), region-4 (containing part of exon 3) and region-5 (containing part of exon 3). Five sets of PCR primers were designed based on the ovine genome sequence (NC_019458.1) for amplification of these regions and their precise coordinates are described in Table 1. These primers were synthesised by Integrated DNA Technologies (Coralville, IA, USA).

Amplifications were performed in a 15 μL reaction containing the purified genomic DNA on one punch of the FTA card, 0.25 μM of each primer, 150 μM of each dNTP (Bioline, London, UK), 2.5 mM of Mg^2+^, 0.5 U of Taq DNA polymerase (Qiagen, Hilden, Germany) and 1 × reaction buffer supplied with the enzyme. The thermal profile for the five regions amplified consisted of 2 min at 94 °C, followed by 35 cycles of 30 s at 94 °C, 30 s at the annealing temperatures shown in Table 1 and 30 s at 72 °C, with a final extension of 5 min at 72 °C. Amplification was carried out in S1000 thermal cyclers (Bio-Rad, Hercules, CA, USA).

The amplicons produced were visualized by electrophoresis in 1% agarose (Quantum Scientific, Queensland, Australia) gels, using 1 × TBE buffer (89 mM Tris, 89 mM Boric acid, 2 mM Na_2_EDTA), containing 200 ng/mL of ethidium bromide. They were then subjected to SSCP analysis. A 0.7 μL aliquot of each amplicon was mixed with 7 μL of loading dye (98% formamide, 10 mM EDTA, 0.025% bromophenol blue, 0.025% xylene-cyanol). After denaturation at 95 °C for 5 min, the samples were cooled rapidly on wet ice and loaded onto 16 cm × 18 cm, 12 or 14% acrylamide:bisacrylamide (37.5:1) (Bio-Rad) gels. Electrophoresis was performed using Protean II xi cells (Bio-Rad) for 19 h in 0.5 × TBE using the conditions described in Table 1. The gels were silver-stained by the method of Byun *et al*. [16].

Amplicons that were identified as potentially homozygous by PCR-SSCP analysis were directly sequenced at the Lincoln University DNA Sequencing Facility. Those DNA sequences only found in a heterozygous form were analysed using an approach that has been described previously [17]. Briefly, a band corresponding to the allele was excised as a gel slice from the polyacrylamide gel, macerated, and then used as a template for re-amplification with the original primers. This second amplicon was then sequenced.

### 2.3. Sequence Analyses

The frequency of occurrence of sequence variants in the different regions was calculated using POPGEN version 3.2 (Molecular Biology and Biotechnology Centre, University of Alberta, Canada). Sequence alignments, translations and comparisons were carried out using DNAMAN version 5.2.10 (Lynnon BioSoft, Vaudreuil, Canada). The BLAST algorithm was used to search the NCBI GenBank databases (http://blast.ncbi.nlm.nih.gov/) for homologous sequences.

### 2.4. Haplotype Determination

Samples were genotyped at region-1 and region-3 of ovine *ADIPOQ*. Haplotypes spanning these two regions of the gene were constructed using those samples that were homozygous in either region. For those samples that were heterozygous in both regions, haplotypes could not be determined, as these two regions span over 11 kb and haplotype-specific PCR amplification [18] would be difficult using the DNA polymerase enzyme employed in this study.

**Table 1:** The primer sequences and PCR-SSCP conditions used for analysis of ovine *ADIPOQ*.

Region Amplified	Primer Binding Region ^a^	Primer Sequence (5'–3')	PCR Annealing Temperature	Amplified Size	SSCP Condition
Region-1	198617873_198617893	F: TTCCTGCTTCTGATCTTGACC	58 °C	388 bp	300 V, 14%, 17 °C
	198618239_198618260	R: CAGCCTAGAAATTGAATCAGTC			
Region-2	198627771_198627789	F: ACAGCGTGGATCTGGGTTC	62 °C	390 bp	200 V, 14%, 32.5 °C
	198628140_198628159	R: CACAATTCACTTTCGGCTGC			
Region-3	198628889_198628909	F: GGTCTTCTTGTTCTCTAGGTC	58 °C	391 bp	200 V, 12%, 20 °C
	198629260_198629279	R: TGGTCCACGTTCTGGTTCTG			
Region-4	198629237_198629256	F: TGCTCTTCACCCACGACCAG	58 °C	373 bp	200 V, 14%, 26 °C
	198629583_198629605	R: GTCCTGCGAACATAGTATATC			
Region-5	198629532_198629553	F: GGATTCTGAACATCATTCATTC	58 °C	455 bp	390 V, 14%, 5 °C
	198629967_198629986	R: CAGATGAGTTGGTGGGAGAC			

^a^ The primer binding position is given relative to the ovine genome sequence (NC_019458.1).

**Figure 1:**
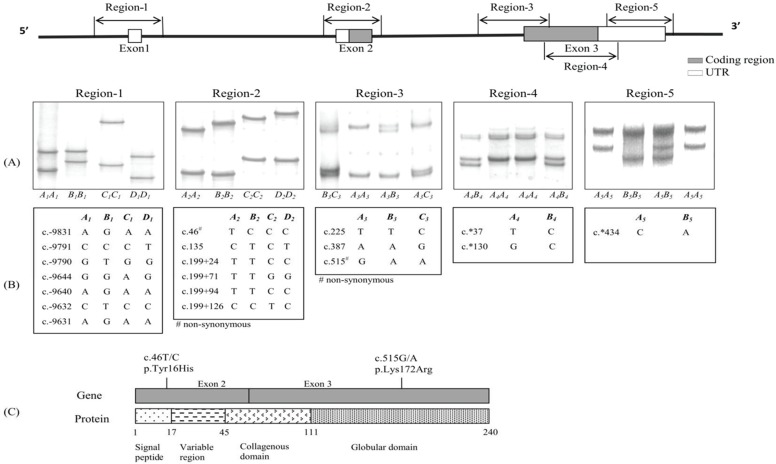
Variation identified in ovine *ADIPOQ*. Unique PCR-SSCP patterns (**A**) representing different DNA sequences (**B**) were detected in region-1 (1); region-2 (2); region-3 (3); region-4 (4); and region-5 (5). Two non-synonymous SNPs that would result in amino acid changes in the signal peptide and the globular domain were identified (**C**). The coordinates of the SNPs are annotated below the patterns based on the ovine whole genome sequence (NC_019458.1) and the numbering of positions follows the guidelines presented at http://www.hgvs.org/mutnomen/.

## 3. Results

### 3.1. Identification of Sequence Variants of Ovine ADIPOQ

Of the 100 samples from the initial five breeds investigated, four SSCP patterns were obtained from the region-1 and region-2 amplicons, three SSCP patterns from the region-3 amplicons, and two SSCP patterns from the region-4 and region-5 amplicons (Figure 1A).

After sequencing, these patterns were confirmed as novel variant sequences of ovine *ADIPOQ* and the sequences were deposited into GenBank with accession numbers as follows: *A_1_-D_1_*: KP903754-KP903757; *A_2_-D_2_*: KP903758-KP903761; *A_3_-C_3_*: KP903762-KP903764; *A_4_-B_4_*: KR9011984-KR011985; and *A_5_-B_5_*: KP903765-KP903766. There were seven, six, three, two and one SNPs identified in region-1, region-2, region-3, region-4 and region-5, respectively (Figure 1B). It is notable that the c.46 T/C substitution in exon 2 putatively results in a non-conservative amino acid change (p.Tyr16His) in the signal peptide, and the c.515A/G substitution in exon 3 putatively results in a conservative amino acid change (p.Lys172Arg) in the globular domain (Figure 1C).

### 3.2. Initial Screen for Variation in ADIPOQ

In the initial 100 samples investigated, polymorphism in region-4 and region-5 was low. There were only two genotypes observed in region-4: *A_4_/A_4_* (91%) and *A_4_/B_4_* (9%) with individual variant frequencies of 95.5% for *A_4_* and 4.5% for *B_4_*; and two genotypes in region-5: *A_5_/A_5_* (89%) and *A_5_/B_5_* (11%) with individual variant frequencies of 94.5% for *A_5_* and 5.5% for *B_5_*. Variants in these two regions were accordingly not investigated in additional sheep.

### 3.3. Frequencies of the Sequence Variants in Different Breeds

In the 316 sheep of various breeds, *A_1_* and *B_1_* (region-1) were the most common variants and observed in all breeds, with an overall frequency of 55.7% and 42.1%, respectively (Table 2). *B_1_* was the most common variant with frequency of 47.3% in Corriedale sheep and *A_1_* was the second most common variant with a frequency of 39.0%. *C_1_* was only observed in Romney sheep, while *D_1_* was observed in Merino, Romney, Corriedale and Perendale sheep, with the lowest overall average frequency of 1.1%.

In region-2, *A_2_* was observed in all of the breeds studied and was the most common variant with an overall frequency of 72.8% (Table 2). Only *A_2_* was observed in the Texel and Dorset Down sheep and *B_2_* was not observed in the Texel, Dorset Down, Romney and Perendale sheep. *B_2_* was the least common variant with an overall frequency of 1.7%. *D_2_* was the second most common variant with an overall frequency of 16.8% and was observed in all of the breeds except Texel and Dorset Down. *C_2_* was observed in all of the breeds, except the Suffolk, Perendale, Texel and Dorset Down sheep.

In region-3, *A_3_* was the most common variant and observed in all of the breeds, with an overall frequency of 49.1% (Table 2). *C_3_* was also found in all breeds with an overall frequency of 46.52%, while *B_3_* was the least common variant with an overall frequency of 4.4%. *B_3_* was not observed in Suffolk, Perendale and Dorset Down sheep.

**Table 2:** Frequencies of ovine *ADIPOQ* variants in various sheep breeds.

Breed	n		Region-1					Region-2					Region-3		
			*A_1_*	*B_1_*	*C_1_*	*D_1_*		*A_2_*	*B_2_*	*C_2_*	*D_2_*		*A_3_*	*B_3_*	*C_3_*
Merino	68		50.0	48.5	0.0	1.5		55.2	5.2	9.6	30.2		66.9	11.8	21.3
Romney	71		52.8	41.6	4.9	0.7		76.8	0.0	4.9	18.3		49.3	1.4	49.3
Suffolk	42		53.6	46.4	0.0	0.0		75.0	1.2	0.0	23.8		35.7	0.0	64.3
Texel	22		56.8	43.2	0.0	0.0		100.0	0.0	0.0	0.0		65.9	6.8	27.3
Corriedale	41		39.0	57.3	0.0	3.7		75.6	2.4	6.1	15.9		28.1	3.7	68.3
Perendale	14		57.1	39.3	0.0	3.6		89.3	0.0	0.0	10.7		42.9	0.0	57.1
Dorper	39		76.9	23.1	0.0	0.0		56.4	1.3	38.5	3.9		53.9	5.1	41.0
Dorset Down	19		81.6	18.4	0.0	0.0		100.0	0.0	0.0	0.0		34.2	0.0	65.8
Overall	316		55.7	42.1	1.1	1.1		72.78	1.7	8.7	16.8		49.1	4.4	46.5

### 3.4. Haplotypes Spanning Region-1 to Region-3

Nine haplotypes that spanned region-1 to region-3 of ovine *ADIPOQ* were identified in the 316 sheep (Table 3). Of these haplotypes, *A_1_-C_3_*, *A_1_-A_3_*, *B_1_-A_3_* and *B_1_-C_3_* were the most common with an overall frequency of 30.1%, 26.4%, 23.2% and 14.9%, respectively. The other five haplotypes were rare, each occurring at frequency of less than 5%.

**Table 3:** *ADIPOQ* haplotypes and frequencies for the region spanning region-1 to region-3.

Haplotype	Frequency	Number (n)
*A_1_-C_3_*	30.1%	139
*A_1_-A_3_*	26.4%	123
*B_1_-A_3_*	23.2%	108
*B_1_-C_3_*	14.9%	69
*B_1_-B_3_*	3.3%	15
*C_1_-A_3_*	1.1%	5
*A_1_-B_3_*	0.7%	3
*C_1_-B_3_*	0.2%	1
*C_1_-C_3_*	0.2%	1

## 4. Discussion

This is the first study to report sequence variation in ovine *ADIPOQ*. Five different regions were investigated in eight commercial sheep breeds from NZ, including meat, wool and dual-purpose breeds. Nineteen SNPs were found suggesting that the ovine gene is quite variable. Extensive heterogeneity has also been described in humans [8,11], pigs [14], cattle [3] and goats [12].

Of the nineteen SNPs detected in this study, only two were non-synonymous. One (c.46T/C) was located in the exon 2 coding region and the other (c.515G/A) was located in the exon 3 coding region. These would potentially result in the amino acid changes p.Tyr16His and p.Lys172Arg, respectively. The substitution p.Tyr16His is in the signal peptide and in the vicinity of the boundary between the signal peptide and variable region, while p.Lys172Arg is in a globular domain of *ADIPOQ* (see Figure 1C). p.Tyr16His may change the structure and thus function of the single peptide, leading to either a change in how the peptide is targeted post-translationally, or a change in the rate or nature of signal peptide processing. It is notable that these two amino acid substitutions occur at amino acid positions that are conserved in many mammalian species including humans, pigs, goats, rabbits, horses and cattle, with Tyr16 and Lys172 being the common residues in these other species.

Two substitutions (rs2241766 G/T in exon 2 and rs17366743 C/T in exon 3) in humans have been reported by Yang *et al*. [19] in the vicinity of ovine c.46T/C and c.515G/A. In humans, these substitutions have been reported to be associated with variation in adiponectin levels in some populations [20]. This would suggest that c.46T/C and c.515G/A are worthy of further investigation in other breeds of sheep including investigation of whether these SNPs are associated with variation in carcass fat traits.

We detected seven SNPs in the ovine *ADIPOQ* promoter, including c.-9831A/G, c.-9791C/T, c.-9790G/T, c.-9644G/A, c.-9640A/G, c.-9632C/T and c.-9631A/G. The effect of these substitutions is difficult to ascertain in the absence of functional studies of the ovine *ADIPOQ* promoter region. It is notable that in the human *ADIPOQ* promoter, multiple physiologically important binding sites have been reported including sites for Sp1, SREBP, AP1 and C/EBP [21]. In humans a proximal substitution (c.-11377C/G) has been reported and this substitution is located in a SP1 binding site [22]. The G nucleotide substitution at c.-11377C/G results in a change in SP1 bonding and causes a reduction in *ADIPOQ* activity [22]. Three other substitutions in the promoter (c.-19166T/G, c.-11426A/G and c.-11391G/A) have also been detected in humans and reported to be associated with type 2 diabetes and obesity [23]. While these SNPs are even further removed from the ovine SNPs described here, it supports the conclusion that additional investigation of promoter region variation is warranted.

In pigs, *ADIPOQ* has been mapped to chromosome 13, and is located near a quantitative trait locus (QTL) for backfat thickness [24] and variation in the average length of the fatty acid chains in the longissimus dorsi muscle [25]. Porcine nucleotide substitutions (c.-90C/A, c.-67G/A and c.-829C/T) have been reported and c.-67G/A and c. -892C/T segregate as two haplotypes (G-C and A-T) that have been associated with carcass and meat quality traits [26].

In cattle, *ADIPOQ* has been mapped to chromosome 1 and is a positional candidate gene for the QTL that affects meat marbling, meat quality grade, ribeye muscle area and weaning weight [27,28]. The SNP c.-176A/G (g.1454) in the bovine *ADIPOQ* promoter has been associated with longissimus dorsi muscle area and backfat thickness, and c.-199C/T (g.1431) and c.-34G/A (g.1596) in the bovine *ADIPOQ* promoter have been associated with fat thickness and ribeye muscle area [3,29].

This evidence from pigs and cattle supports the contention that the variation described here in ovine *ADIPOQ* may affect key sheep traits, including potentially fat deposition, fat composition and meat quality, and possibly also growth traits.

Four of the ovine substitutions c.199+24T/C, c.199+71T/G, c.199+94T/C and c.199+126T/C were found in intron 2. They would be unlikely to affect the structure or function of *ADIPOQ* directly unless they are linked to other variation in coding regions of the gene, or regions that may affect the processing of the primary transcript including its stability or longevity. It is also known that non-coding RNAs transcribed from intronic regions can affect transcription efficiency by affecting regulatory elements, such as enhancers, silencers or other DNA structures [30,31]. In human *ADIPOQ*, intron 2 variation (rs17366743: c.214+276A/C) has been linked with promoter polymorphisms in the gene, including c.-11426A/G, c.-11377C/G and c.-11391G/A, and is associated with *ADIPOQ* levels in diabetes and obesity [20]. In pig, variation in the non-coding regions of *ADIPOQ* has been associated with production traits, with SNP c.178A/G (g.1735) in porcine intron 2 having a significant effect on shoulder fat thickness [13].

We detected three SNPs in the ovine *ADIPOQ* 3'-UTR (c.*37T/C, c.*130G/C and c.*434C/A). In humans, at least ten SNPs in the 3'-UTR of *ADIPOQ* have been described, but most of them are rare. Human SNPs rs1063537 T/C, rs1063539 C/G and rs6773957 A/G are more common and have an association with type 2 diabetes and obesity in a Chinese population [20,32]. In goats, two nucleotide substitutions (c.*88T/A and c.*210A/G) have been described in the 3'-UTR. The relationships between these two SNPs and body weight at different ages, has been analysed, but no associations were found [33].

In this work, different frequencies were found for ovine *ADIPOQ* variants in different breeds. The different origins of these breeds may be reflected in the variant frequencies found in *ADIPOQ*. The majority of NZ sheep breeds are derived from European breeding stock. For example, the difference in variant frequencies in Romney and Perendale breeds in this study was small. This is probably because the Perendale breed was developed from a Romney-Cheviot cross. However, although the Corriedale sheep was developed by crossing Lincoln rams with Merino ewes, the variant frequencies in the Corriedale and Merino sheep are different. This may be a consequence of the foundation sheep for the Corriedale breed having different variant frequencies than the base Merino population.

Haplotypes can span a large region of the gene and help increase the Polymorphism Information Content (PIC). In this context, *A_1_* and *B_1_* were the most common variants (55.70% and 42.09%) in region-1; *A_2_* was the most common (72.78%) in region-2; and *A_3_* and *C_3_* were the most common variants (49.05% and 46.52%) in region-3. This prompted us to undertake further analysis of region-1 and region-3. Nine haplotypes were observed spanning the two regions. Only *A_1_-A_3_*, *A_1_-C_3_*, *B_1_-A_3_* and *B_1_-C_3_* were common. Due to the promoter (region-1) and intron 2-exon 3 region (region-3) of *ADIPOQ* playing an important role in regulating *ADIPOQ* function in humans, these regions and haplotypes may be worthy of further investigation in sheep. More haplotypes may also be found when more of the gene is analysed in larger numbers of sheep.

In summary, the work presented in this study suggests that ovine *ADIPOQ* has high levels of polymorphism in sheep, and it could be speculated that even more new variants will be found when more samples and different breeds are analysed. Given the apparently important role of *ADIPOQ* in metabolism, we can also speculate that some of the genetic variation described in this study may be associated with sheep carcass traits. However, because only a small number of sheep were investigated here a much larger association study will be required.

## 5. Conclusions

This study used PCR-SSCP to screen for variation in ovine *ADIPOQ* and identified a total of 19 SNPs. The majority of these were found in non-coding regions. There were nine haplotypes of *ADIPOQ* identified and the frequencies of these haplotypes varied in different breeds of sheep. The effect of this variation in ovine *ADIPOQ* on sheep production traits warrants investigation.
